# *Helicobacter pylori* and Pathogenesis

**DOI:** 10.1155/2015/304768

**Published:** 2015-04-08

**Authors:** Akio Tomoda, Shigeru Kamiya, Hidekazu Suzuki

**Affiliations:** ^1^Tokyo Medical University, Tokyo 160-8402, Japan; ^2^Kyorin University School of Medicine, Mitaka 181-8611, Japan; ^3^Keio University School of Medicine, Tokyo 160-8582, Japan

By calling for manuscripts for this special issue, many manuscripts were submitted to the editorial office. After careful reviewing by expert referees, highly qualified papers concerning the topics were accepted as review and research articles for publication in the journal. From the accepted articles, some interesting ones are introduced as follows.

S. K. Pachathundikandi et al. reviewed an interplay of* H. pylori* with toll-like receptors (TLRs). TRL2 is able to recognize various different pathogen associated molecular patterns (PAMPs) including lipoproteins, lipoteichoic acid, and peptidoglycan.* H. pylori* activated NF-*κ*B primarily through TLR2 and induced chemokine expression. Lipopolysaccharide (LPS) of* H. pylori* was identified as the ligand for TLR4, and* H. pylori* induced the secretion of IL-12 and IL-10 in mouse macrophages through TLR4/MyD88. It was also shown that* H. pylori* LPS can promote proliferation and progression of gastric cancer cells via a TLR4-dependent pathway. Flagellin from* H. pylori* is the ligand for TLR5, and the involvement of TLR5 in the recognition and further inflammatory processes is important for establishing a persistent infection of* H. pylori* at the mucosal surface. A chimeric flagellin composed of terminal regions from* Escherichia coli* and the middle region from* H. pylori* was reported to activate TLR5, suggesting that the chimeric flagellin might be a vaccine candidate with significant protection against* H. pylori* infection. Correlation between TLR8/9 sensing nucleic acids and* H. pylori *infection is also discussed in the review article.

T. Nishizawa and H. Suzuki reviewed recent findings on gastric carcinogenesis and underlying molecular mechanisms. Reactive oxygen species (ROS) induced by* H. pylori* can bind with nucleic acids, turning them into mutated forms that play a role in multistep carcinogenesis. Correlation of CD44 variant, cell-surface marker of cancer stem-like cells with ROS defense system was reported. The important roles of CagA and activation-induced cytidine deaminase (AID) in carcinogenesis are also reviewed.* H. pylori* infection up- or downregulates expression of microRNAs that is linked to gastric tumorigenesis. Activation of epidermal growth factor receptor (EGFR) and erythroblastic leukemia-associated viral oncogene B (ErbB2) induced by* H. pylori* infection results in survival of gastric epithelial cells with DNA damage. In addition, recent advances in molecular targeting therapies by anti-EGRF are introduced.

H. Tsugawa et al. identified novel FecA1-binding compounds* in silico* and examined the effect of NDGA (nordihydroguaiaretic acid) that is one of the above compounds, on SodB activity, metronidazole (Mtz) susceptibility, and H_2_O_2_ sensitivity of* H. pylori.* NDGA reduced SodB activity and increased both H_2_O_2_ sensitivity and Mtz susceptibility. These results suggest that NDGA might be effective for the development of a novel eradication therapy.

Y. Shan et al. reported that outer membrane protein 18 (Hp1125) of* H. pylori* is involved in persistent colonization by evading interferon- (IFN-) gamma signaling. It was shown that IFN-gamma induced higher expression of* H. pylori* Omp18 and reduced the expression of CagA and NapA. By mouse infection model, isogenic omp18 mutant strain showed defective colonization and increased inflammatory changes in gastric mucosa. It was also shown that the isogenic mutant strain induced more production of cytokine, chemokine, and NO, indicating that Omp18 is involved in bacterial survival against oxidative stress and phagocytosis by macrophages. Comment on this paper was sent from A. T. B. Abadi and E. Ierardi. They hypothesize that more factors except Omp18 are contributing to long term infection of* H. pylori *in gastric mucosa as the connection of a unique factor to the drive of the final pattern of this phenomenon could be too speculative.

O. Feliciano et al. reported the prevalence of* vacA*,* cagA,* and* iceA *genotypes of* H. pylori* strains isolated from Cuban patients with upper gastrointestinal diseases. It was shown that the* vacA *s1 allele,* cagA* gene, and* iceA2 *allele were the most prevalent (72.0%, 56.0%, and 57.3%, resp.). Significant statistical association was observed between* iceA2 *allele and patients with nonpeptic ulcer dyspepsia as well as virulence genotypes (*s1, s1m2*) and patients over 40 years old. Although the total number (*n* = 75) of the isolates was not enough to conclude clearly, it was indicated that a high prevalence of main virulence factors was detected in Cuban isolates similar to that observed in other Western populations.

Since the discovery of* H. pylori *in 1983 (first isolation in 1982), many research studies were performed to clarify the mechanisms by which this microorganism causes not only gastroduodenal diseases including gastric cancer but also extragastric diseases such as idiopathic thrombocytopenic purpura and iron-deficiency anemia. However, the details on the correlation between* H. pylori* infection and gastric/extragastric pathogenesis in human remain to be determined. The review and research articles published in this special issue may give us a hint to resolve the above question, but further studies on pathogenesis of* H. pylori* infection need to continue to be done.

*Akio Tomoda* *Shigeru Kamiya* *Hidekazu Suzuki*

